# The Impact of Clinical Information on the Assessment of Endoscopic Activity: Characteristics of the Ulcerative Colitis Endoscopic Index Of Severity [UCEIS]

**DOI:** 10.1093/ecco-jcc/jjv077

**Published:** 2015-05-08

**Authors:** Simon P. L. Travis, Dan Schnell, Brian G. Feagan, Maria T. Abreu, Douglas G. Altman, Stephen B. Hanauer, Piotr Krzeski, Gary R. Lichtenstein, Philippe R. Marteau, Jean-Yves Mary, Walter Reinisch, Bruce E. Sands, Patrick Schnell, Bruce R. Yacyshyn, Jean-Frédéric Colombel, Christian A. Bernhardt, William J. Sandborn

**Affiliations:** ^a^Translational Gastroenterology Unit, John Radcliffe Hospital, Oxford, UK; ^b^Middletown, OH, USA; ^c^Robarts Research Institute, University of Western Ontario, London, ON, Canada; ^d^Division of Gastroenterology, University of Miami Leonard M. Miller School of Medicine, Miami, FL, USA; ^e^Centre for Statistics in Medicine, University of Oxford, Oxford, UK; ^f^Digestive Disease Center, Northwestern University Feinberg School of Medicine, Chicago, IL, USA; ^g^Medpace, Warsaw, Poland; ^h^Division of Gastroenterology, Department of Medicine, University of Pennsylvania, Philadelphia, PA, USA; ^i^AP-HP, Hôpital Lariboisière Medicosurgical Department of Digestive Diseases and University Denis Diderot, Paris, France; ^j^INSERM U717 Biostatistics and Clinical Epidemiology, Université Paris Diderot, Paris, France; ^k^Division of Gastroenterology, Department of Medicine, McMaster University, Hamilton, ON, Canada; ^l^Dr. Henry D. Janowitz Division of Gastroenterology, Icahn School of Medicine at Mount Sinai, New York, NY, USA; ^m^University of Minnesota, Minneapolis, MN, USA; ^n^Division of Digestive Diseases, University of Cincinnati, Cincinnati, OH, USA; ^o^Hôpital Claude Huriez, Centre Hospitalier Universitaire de Lille, Lille, France; ^p^Bernhardt Regulatory Consulting, Cincinnati, OH, USA; ^q^Division of Gastroenterology, University of California San Diego, La Jolla, CA, USA

**Keywords:** Endoscopic score, ulcerative colitis, disease activity index

## Abstract

**Background and Aims:**

To determine whether clinical information influences endoscopic scoring by central readers using the Ulcerative Colitis Endoscopic Index of Severity [UCEIS; comprising ‘vascular pattern’, ‘bleeding’, ‘erosions and ulcers’].

**Methods:**

Forty central readers performed 28 evaluations, including 2 repeats, from a library of 44 video sigmoidoscopies stratified by Mayo Clinic Score. Following training, readers were randomised to scoring *with* [‘unblinded’, *n* = 20, including 4 control videos with misleading information] or *without* [‘blinded’, *n* 20] clinical information. A total of 21 virtual Central Reader Groups [CRGs], of three blinded readers, were created. Agreement criteria were pre-specified. Kappa [κ] statistics quantified intra- and inter-reader variability.

**Results:**

Mean UCEIS scores did not differ between blinded and unblinded readers for any of the 40 main videos. UCEIS standard deviations [SD] were similar [median blinded 0.94, unblinded 0.93; *p* = 0.97]. Correlation between UCEIS and visual analogue scale [VAS] assessment of overall severity was high [r blinded = 0.90, unblinded = 0.93; *p* = 0.02]. Scores for control videos were similar [UCEIS: *p* ≥ 0.55; VAS: *p* ≥ 0.07]. Intra- [κ 0.47–0.74] and inter-reader [κ 0.40–0.53] variability for items and full UCEIS was ‘moderate’-to-‘substantial’, with no significant differences except for intra-reader variability for erosions and ulcers [κ blinded: 0.47 vs unblinded: 0.74; *p* 0.047]. The SD of CRGs was lower than for individual central readers [0.54 vs 0.95; *p* < 0.001]. Correlation between blinded UCEIS and patient-reported symptoms was high [stool frequency: 0.76; rectal bleeding: 0.82; both: 0.81].

**Conclusions:**

The UCEIS is minimally affected by knowledge of clinical details, strongly correlates with patient-reported symptoms, and is a suitable instrument for trials. CRGs performed better than individuals.

## 1. Introduction

Endoscopy is central to the assessment of disease activity in ulcerative colitis [UC] both in trials and in clinical practice, but assessment is often inconsistent.^1,2,3,4,5^ Activity indices for UC, such as the Mayo Clinic Score, incorporate an endoscopic component and are commonly used to evaluate response to treatment in clinical trials.^6^ Lack of consistency can affect the outcomes of trials, independently of the effect of treatment, and negatively affect decisions by regulatory authorities or clinicians.^7^ The Ulcerative Colitis Endoscopic Index of Severity [UCEIS] was developed as a rigorous instrument for assessing endoscopic disease activity in UC.^5,8^

The UCEIS was developed using a predefined protocol. Initially, the level of disagreement for 10 endoscopic items, each with 3–5 levels of severity, was determined among 10 investigators.^5^ The intra- and inter-investigator variability for each item was then assessed by 30 different investigators, and a model constructed that best represented overall endoscopic activity evaluated on a visual analogue scale [VAS]. The final model consisted of three items: vascular pattern [3 levels], bleeding [4 levels], and erosions and ulcers [4 levels] [Table 1].^5,8^ In practice, the UCEIS is scored from the worst affected area at video sigmoidoscopy and the final score is the sum of components ranging from 0 [normal] to 8 [most severe; it should be noted that this simplifies the original index which ranged from 3–11].^5,8^ The UCEIS accounted for 88% of the variance between observers in the overall assessment of endoscopic activity in the test cohort^5^ and 86% in the validation cohort.^8^

**Table 1.** The UCEIS: items, levels, and definitions used as anchor points for evaluating ulcerative colitis.^5,8
^

Descriptor [score most severe lesions]	Likert scale anchor points	Definition
Vascular pattern	Normal [0]	Normal vascular pattern with arborisation of capillaries clearly defined, or with blurring or patchy loss of capillary margins
	Patchy obliteration [1]	Patchy obliteration of vascular pattern
	Obliterated [2]	Complete obliteration of vascular pattern
Bleeding	None [0]	No visible blood
	Mucosal [1]	Some spots or streaks of coagulated blood on the surface of the mucosa ahead of the scope that can be washed away
	Luminal mild [2]	Some free liquid blood in the lumen
	Luminal moderate or severe [3]	Frank blood in the lumen ahead of endoscope or visible oozing from mucosa after washing intra-luminal blood, or visible oozing from a haemorrhagic mucosa
Erosions and ulcers	None [0]	Normal mucosa, no visible erosions or ulcers
	Erosions [1]	Tiny [< 5 mm] defects in the mucosa, of a white or yellow colour with a flat edge
	Superficial ulcer [2]	Larger [> 5 mm] defects in the mucosa, which are discrete fibrin-covered ulcers when compared with erosions, but remain superficial
	Deep ulcer [3]	Deeper excavated defects in the mucosa, with a slightly raised edge

The worst affected area of the colon visible at sigmoidoscopy is scored. The copyright of UCEIS is held by Watson Laboratories, a subsidiary of Actavis Inc., as successor in interest of Warner Chilcott and Procter and Gamble.

The aim of the present study was to understand the UCEIS in a clinical context. The primary objective was to determine whether the UCEIS is affected by clinical information, using an independent cohort of central reader investigators. Henceforth, the term ‘readers’ rather than ‘investigators’ or ‘observers’ will be used for consistency, since ‘investigators’ implies clinicians who recruit patients to trials and often have knowledge of symptoms, whereas ‘readers’ implies independence from the patient, and ‘observers’ is a generic term for either. Secondary objectives were to investigate potential benefits of a group of central readers for reducing variability,^9^ and to compare scores with other indices and patient-reported symptoms.

## 2. Materials and Methods

### 2.1. Development of the UCEIS

The development of the UCEIS has been reported.^5,8^ In short, the resource was a library of 670 videosigmoidoscopies from patients in three clinical trials, supplemented by individuals without UC and hospitalised patients with acute severe UC.^5^ In phase 1, the authors determined agreement in overall endoscopic assessment and defined descriptive terms [‘items’]. Phase 2, conducted in a separate cohort of 30 investigators, rated items in 25 of 60 different videos and assessed overall severity on a 100-point VAS [0 completely normal and 100 = worst ever seen].^8^ The UCEIS developed from this study included three items [Table 1].^5,8^ The UCEIS is freely accessible to all at no cost, though the terminology is subject to copyright and acknowledgement. Phase 3, in another cohort of 25 investigators, demonstrated reproducibility of the UCEIS in 28 of 57 videos and rebased normality to ‘0’, rather than ‘3’.^8^ This paper reports phase 4, to evaluate the effect of clinical information on endoscopic scoring.

### 2.2. Assessment of the impact of knowledge of clinical information on UCEIS scoring

#### 2.2.1. Investigators

A new cohort of 40 investigators [‘readers’] was enrolled from Europe, North America and Australasia, all of whom were experienced in evaluating endoscopies in patients with UC. Three had previously participated in phase 2 or 3 of UCEIS development and all were blinded to the objectives of the study. To investigate the effect of clinical information on UCEIS scoring, investigators were alternately assigned to the blinded [*n* = 20] and unblinded [*n* = 20] assessment groups as they completed the study enrolment requirements [but prior to training or receipt of any study videos]. All underwent training on the UCEIS, which excluded clinical information and involved scoring four standard videos; to qualify [details in 3. Results], assessments on each video had to align with the level of ‘erosions and ulcers’ assigned by the authors [SPLT,PK,BRY,WJS], and within one level for vascular pattern and bleeding. For readers initially failing to qualify, a retest was permitted; they had correctly to score two of three different videos for the item[s] that they had previously scored incorrectly. Readers who failed the second qualifier were excluded.

#### 2.2.2. Video selection

A new library of 44 anonymised videos was created from 670 videos and supplements created for phase 1–3, stratified by clinical disease activity [Mayo Clinic Score; MCS] assigned on the date that they were derived. [According to MCS: stool frequency: 0 = normal, 1 = 1 to 2 more stools than normal, 2 = 2 to 3 more stools than normal, 3 = >4 more stools than normal; rectal bleeding: 0 = none, 1 = visible blood with stool < half the time, 2 = visible blood with stool > half the time, 3 = passing blood alone; Physician’s Global Assessment: 0 = normal, 1 = mild, 2 = moderate, 3 = severe.]

A total of 34 of the videos were selected [by PK and BRY] from sigmoidoscopies conducted to a standard procedure as part of clinical trials.^10,11^ No video had been used in the earlier phases of UCEIS development. Three further videos were taken from patients with severe UC [recorded before colectomy] and three from subjects without UC [colorectal cancer screening]. Four videos were repeated as common controls between readers, drawn from the 34 videos of patients with active disease [two videos with MCS 1–2 and two with MCS 10–11].

#### 2.2.3. Video allocation

**Figure 1.**
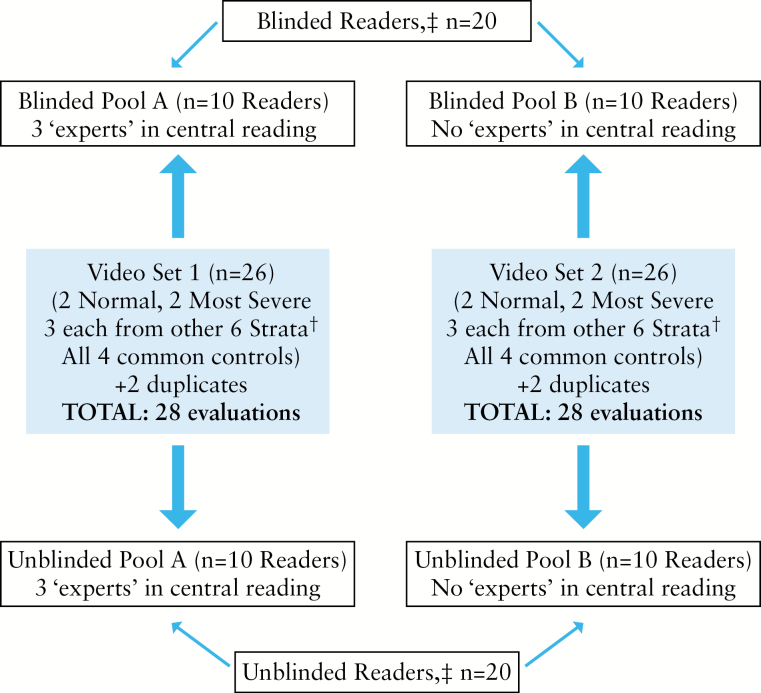
Schematic of study design. ‡Unblinded readers were provided with clinical disease activity information [symptoms and history] relating to each video, whereas blinded readers were not. †Strata: Mayo Clinic score [MCS] 0; MCS 1–2; MCS 3–5; MCS 6–7; MCS 8–9, MCS 10–11. One normal video, one most severe video, and one Mayo score 0 video were part of both pools.

Each reader performed 28 evaluations from the 44 videos, which included two repeats of non-control videos to evaluate intra-reader variation and the four common controls [Figure 1]. Readers were either provided with clinical information on disease activity for each video [one or two sentences on age, symptoms and history, summarised by PK/BRY/ST; unblinded group] or with no accompanying clinical information [blinded group]. Patient information was extracted from the original trials.^10,11^

To ensure some disparity between clinical information and endoscopic assessment, the two common control videos with MCS 1–2 in the unblinded group were assigned information more severe than reported [originally: rectal bleeding [RB] = 1 / stool frequency [SF] = 0 / Physician’s Global Assessment [PGA] = 0; and RB = 0/SF = 1/PGA = 0; both changed to: RB = 1/SF = 2/PGA = 1]. The two control videos with MCS 10–11 were given symptom information less severe [originally: RB = 3/SF = 3/PGA = 3; and RB = 2/SF = 3/PGA = 3; both changed to: RB = 2/SF = 2/PGA = 2].^1^ Readers in the blinded group were not provided with any clinical information for the common control videos, as with their other videos.

To ensure that sufficient numbers of readers viewed the same videos to power the analyses, two ‘pools’ of 26 videos [*n* = 28, including the two duplicates] were created with 10 unblinded and 10 blinded readers randomly allocated to each pool.

#### 2.2.4. Video evaluation

The 40 readers who passed training evaluated the UCEIS [Table 1] in the worst area affected at videosigmoidoscopy. As with phase 3,^8^ still photographs from the training were provided for reference during the evaluations and the overall assessment of endoscopic severity recorded on a 100-point VAS [0 = completely normal and 100 = worst ever seen]. Data were captured using a programme developed by one of the authors [PS] that ran simultaneously and saved responses after scoring each video.

### 2.3. Assessment of the potential for central reader groups to reduce overall variability

The effect of central reading to improve consistency of scoring the UCEIS was examined through ‘virtual’ central reading groups [CRGs] of three readers. Some of the readers [6/40] were recruited specifically for their experience as central readers in other trials [see acknowledgements]; three were randomly allocated to the blinded group and three to the unblinded group [Figure 1]. In the blinded group, each CRG consisted of one randomly chosen ‘experienced central reader’, together with two randomly selected ‘standard readers’ from the other seven in the group [all, therefore, scored the same videos]. This allowed 21, 3-person virtual CRGs to be created, representing 21 possible pairings of two ‘standard readers’ with one of three ‘experienced central readers’. Thus, each virtual CRG differed by at least one standard reader, to maximise independence. Adjudication of the UCEIS within the virtual CRG was accomplished as follows: if the UCEIS score was agreed between the two ‘standard’ blinded readers, then that score was considered the adjudicated CRG score; if their UCEIS scores did not agree, then the score from the ‘experienced central reader’ was included and the adjudicated CRG UCEIS score was set to the median of the three. Such detail matters when considering the implications of central reading.^9^

### 2.4. Statistical considerations

#### 2.4.1. Calculating the UCEIS

The UCEIS represents the simple sum of ‘vascular pattern’ [scored 0 to 2], ‘bleeding’ [0 to 3] and ‘erosions and ulcers’ [0 to 3]. Thus, UCEIS scores range from 0 [normal] to 8 [most severe].

#### 2.4.2. Primary objective: impact of clinical information on UCEIS scoring

UCEIS and overall severity by VAS [0–100] for each of the 40 videos in the main analysis dataset were compared between the blinded and unblinded groups using the Wilcoxon rank sum test [exact *p*-value with Holm’s multiplicity adjustment^12^]. Standard deviations [SD] of the assessments on a per video basis were compared using the Wilcoxon signed rank test. The correlation between the UCEIS and overall endoscopic assessment of severity by VAS, as quantified by Pearson correlation coefficients, was compared between the blinded and unblinded groups. Comparison of these correlations allowed the accuracy of UCEIS assessments to be checked, in addition to quantifying the similarity of accuracy estimates between the blinded and unblinded groups. Pearson correlation coefficients were calculated from reader’s scores of the UCEIS for their set of videos and the mean VAS for the appropriate videos, derived from the responses of all other readers in the blinded or unblinded group. This addressed any lack of independence between UCEIS and evaluation of overall severity. Correlations were summarised by median, minimum, and maximum within each group, and compared using the Wilcoxon rank sum test. The UCEIS and overall [VAS] severity scores for the common control videos, which were presented with misleading symptom information to the unblinded group, were also compared using the Wilcoxon rank sum test.

Intra- and inter-reader variability in blinded and unblinded groups was evaluated through kappa [κ] statistics, as qualitatively interpreted by Landis and Koch [κ: < 0.00, ‘poor’ agreement; 0.00–0.20, ‘slight’ agreement; 0.21–0.40, ‘fair’ agreement; 0.41–0.60, ‘moderate’ agreement; 0.61–0.80, ‘substantial’ agreement; 0.81–1.00, ‘almost perfect’ agreement].^13^ The standard kappa summarised the precise level of agreement, used for individual items. Since the overall UCEIS score is a 9-point [0–8] ordinal scale, a weighted kappa was also calculated to take account of close agreement, assigning a weight of 1 for precise agreement, 0.5 for scores that differed by 1 level, and 0 in all other cases. For intra-reader variability analyses, only data from duplicate videos were used. Inter-reader κ values were calculated by stratifying by reader pairs and using the common videos that they scored, but excluding second scoring of duplicate videos. An average of reader-pair κ values [‘overall κ’] was calculated, where the weighting was the inverse of their variance.

#### 2.4.3. Secondary objective: assessment of the potential benefits of CRGs in reducing variability

To evaluate the effect of blinded central reader groups, for the 18 non-control videos read by all three experienced central readers, SDs were calculated across the seven blinded ‘standard readers’ and compared with those across the 21 virtual CRGs. Differences in SDs between groups were assessed by nonparametric methods.

#### 2.4.4. Secondary objective: comparison of UCEIS with other indices and patient-reported symptoms

The comparative indices selected were the Mayo Clinic Score [MCS],^14^ partial MCS [excluding endoscopic subscore],^15^; and the modified Baron score,^14^ as recorded in the original clinical trials. To compare the UCEIS with patient-reported symptoms and variables recorded in practice, stool frequency [SF], rectal bleeding [RB], both SF and RB, and patient functional assessment were derived from subscores of the MCS contemporaneously recorded for each video. These variables were compared with UCEIS scores for the blinded readers, excluding those for ‘normal’ and ‘most severe’ videos [for which there was no MCS] and common controls [which were repeats], by Spearman rank correlation. SF, RB, PFA, and partial MCS were also correlated with the modified Baron score, to determine how results compared with those calculated for the UCEIS. The relationship between the UCEIS bleeding item [scored 0–3] and patient-reported RB on a 3-point scale [0 = none to 2 = visible blood with stool > half the time or passing blood alone] was also evaluated. Finally, the distribution of RB, SF, and RB and SF items across UCEIS scores 0–8 was assessed for blinded video evaluations, excluding ‘normal’, ‘most severe’, and common control videos.

Except as noted for comparison of mean scores by video, statistical significance was assumed at the 5% level [unadjusted *p* < 0.05], using the Statistical Analysis System [SAS, Cary, NC] software, version 9.2.

### 2.5. Ethical statement

All subjects had consented for anonymised presentation of their video sigmoidoscopies [EUDRACT 2006-001310-32; Oxford LREC 536407Q1605/58ORH].

## 3. Results

### 3.1. Reader qualification

A total of 47 readers underwent UCEIS training to reach the target of 40 for the study. Of those that qualified, 20 succeeded on their initial assessment and the remainder after scoring the additional set of three videos. The remaining seven physicians failed to qualify. Variance between qualifiers was not evaluated, because of disparity between reader numbers and variables.

### 3.2. Video evaluation

A total of 1120 evaluations were performed across the 44 videos [40 readers x 28 evaluations]. The main analysis dataset consisted of 880 evaluations [440 each for blinded and unblinded groups] across 40 videos, obtained by removing evaluations of the four common control videos and the second evaluation in each of the two repeated videos. The dataset for the four common control videos consisted of 160 evaluations [40 readers x 4 videos]. There were also 160 evaluations in the dataset for assessing intra-reader variability [40 readers x 2 repeat pairs].

### 3.3. Range of disease severity

**Figure 2.**
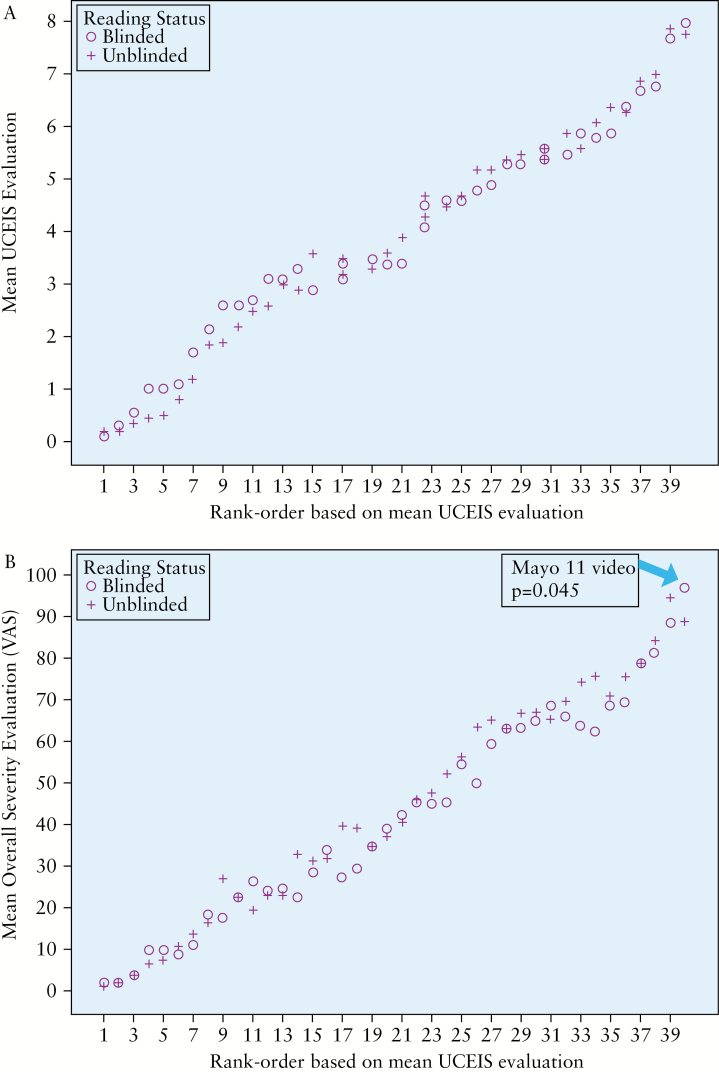
Mean blinded and unblinded UCEIS [A] and VAS [B] scores for the main evaluation set of 40 videos.

Mean assessments of overall endoscopic severity by VAS [0–100] ranged from 1.65 for videos in the ‘normal’ stratum to 92.75 for videos in the ‘most severe ever seen’ stratum according to the 40 readers. Using the same videos, this corresponded to mean UCEIS scores of 0.15/8 for ‘normal’ subjects to 7.90/8 for ‘most severe’, indicating that the videos comprehensively covered the range of endoscopic severity of UC [Figure 2].

### 3.4. Impact of knowledge of clinical information on UCEIS scoring

#### 3.4.1. Mean agreement, variability, and correlation in UCEIS and overall severity scores

**Figure 3.**
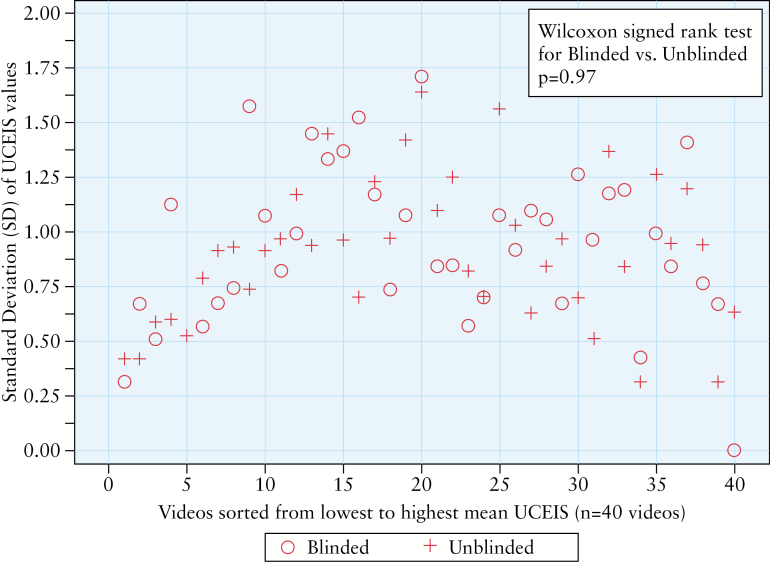
Standard deviations for blinded and unblinded groups for the main evaluation set of 40 videos.

Mean UCEIS scores did not differ between blinded and unblinded readers for any of the 40 videos in the main analysis set [Wilcoxon rank sum tests with Holm’s multiplicity adjustment, all *p*-values ≥ 0.05] [Figure 2A]. There was one video [severe UC recorded before colectomy] for which the VAS score was significantly higher in the blinded group [*p* = 0.045] [Figure 2B]. There were no systematic differences found in the UCEIS SDs between the blinded and unblinded groups in the main dataset [median SD 0.94 vs 0.93, respectively; *p* = 0.97], although as expected, the SD was lowest at the severe end of the video spectrum [Figure 3]. Median correlation between UCEIS and overall severity on the VAS was high in both the blinded [median = 0.899, minimum = 0.80, maximum = 0.967] and unblinded [median = 0.933, minimum = 0.856, maximum = 0.972] groups in the main dataset of 40 videos. The difference between the median correlations, though small [0.034], was statistically significant [*p* = 0.02]. For the four common control videos, there were no statistically significant differences in UCEIS [*p* ≥ 0.55] or overall severity scores [*p* ≥ 0.07] between reader groups.

#### 3.4.2. Intra-reader and inter-reader agreement

Overall, intra-reader variability for the three items ranged from κ 0.47 to 0.74, indicating ‘moderate’ to ‘substantial’ agreement [Table 2]. Intra-reader agreements for ‘vascular pattern’ and ‘bleeding’ items were similar for the blinded and unblinded groups, whereas the difference for the ‘erosions and ulcers’ item [blinded: κ 0.47 vs unblinded: κ 0.74; *p* = 0.047] just reached statistical significance. Clinical information tended to increase variability [i.e. reduced the κ] for the bleeding item and improved consistency between readers for erosions and ulcers. Weighted intra-reader kappas for the full UCEIS [sums of the three descriptors] were 0.51 (95% confidence interval [CI] = 0.36, 0.66) and 0.56 [95% CI = 0.42, 0.69] for the blinded and unblinded readers, respectively, and were not significantly different [*p* = 0.66].

**Table 2.** Intra-reader agreement [κ] for UCEIS items in blinded and unblinded groups.

Descriptor	Blinded readers [*n* = 20] [95% CI]	Unblinded readers [*n* = 20] [95% CI]	*p*-value for difference between κ
Vascular pattern [0–2]	0.57 [0.34, 0.80]	0.62 [0.41, 0.84]	0.72
Bleeding [0–3]	0.68 [0.49, 0.87]	0.47 [0.25, 0.69]	0.15
Erosions and ulcers [0–3]	0.47 [0.26, 0.68]	0.74 [0.57, 0.91]	0.047
UCEIS [0–8] +	0.51 [0.36, 0.66]	0.56 [0.42, 0.69]	0.66

A total of 80 repeat-pair evaluations assessed intra-reader variability [160 evaluations in total]. κ: < 0.00, ‘poor’ agreement; 0.00–0.20, ‘slight’ agreement; 0.21–0.40, ‘fair’ agreement; 0.41–0.60, ‘moderate’ agreement; 0.61–0.80, ‘substantial’ agreement; 0.81–1.00, ‘almost perfect’ agreement.^13^ + Weighted kappa [weight of 1 for perfect agreement; 0.5 for difference in level of 1; and 0 otherwise].

Inter-reader agreement for the items was also ‘moderate’ to ‘substantial’ [κ 0.40 to 0.71; Table 3]. There were no significant differences in inter-reader variability between blinded and unblinded groups for any of the items, whether analysed within the 40 videos in the main dataset [excluding the four common control videos and the second evaluation in each of the two repeated videos] or across the four common control videos. Weighted inter-reader kappas for the full UCEIS were 0.47 [95% CI = 0.46, 0.49] and 0.47 [95% CI = 0.44, 0.50] for the blinded and unblinded readers, respectively.

**Table 3:** Inter-reader agreement [κ] for UCEIS items in blinded and unblinded groups

Item	Main analysis dataset^a^			Common control dataset^b^		
	Blinded readers [*n* = 20] [95% CI]	Unblinded readers [*n* = 20] [95% CI]	*p*-value for difference between κ	Blinded readers [*n* = 20] [95% CI]	Unblinded readers [*n* = 20] [95% CI]	*p*-value for difference between κ
Vascular pattern [0–2]	0.50 [0.46, 0.54]	0.53 [0.48, 0.58]	0.33	0.67 [0.62, 0.71]	0.66 [0.62, 0.70]	0.97
Bleeding [0–3]	0.40 [0.36, 0.43]	0.44 [0.41, 0.47]	0.06	0.55 [0.51, 0.58]	0.56 [0.52, 0.59]	0.93
Erosions and ulcers [0–3]	0.48 [0.45, 0.52]	0.47 [0.44, 0.50]	0.62	0.71 [0.67, 0.74]	0.68 [0.64, 0.71]	0.87
UCEIS [0–8] +	0.47 [0.46, 0.49]	0.47 [0.44, 0.50]	0.85	0.55 [0.52, 0.58]	0.54 [0.51, 0.57]	0.61

^a^A total of 880 evaluations [440 each for blinded and unblinded groups] across 40 videos, obtained by removing evaluations of the 4 common control videos and the second evaluation in each of the 2 repeated videos.

^b^A total of 160 evaluations [40 readers x 4 videos]. κ: < 0.00, ‘poor’ agreement; 0.00–0.20, ‘slight’ agreement; 0.21–0.40, ‘fair’ agreement; 0.41–0.60, ‘moderate’ agreement; 0.61–0.80, ‘substantial’ agreement; 0.81–1.00, ‘almost perfect’ agreement^13^ + Weighted kappa [weight of 1 for perfect agreement; 0.5 for difference in level of 1; and 0 otherwise].

### 3.5. Potential benefits of central reader groups to reduce overall variability

**Figure 4.**
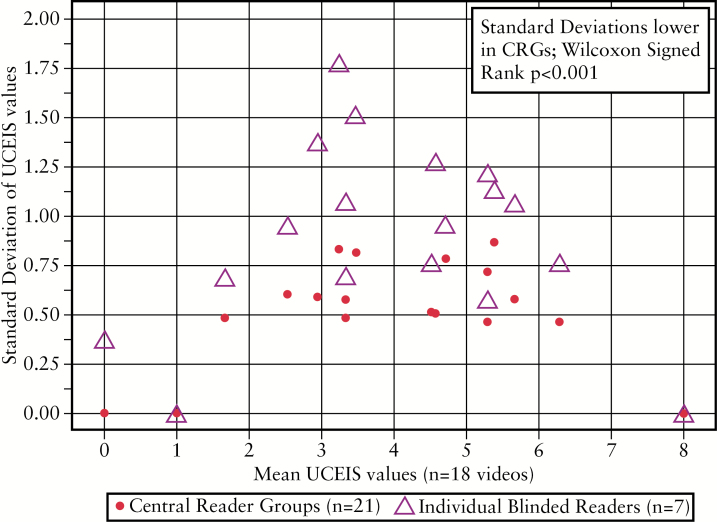
Standard deviations for the blinded, virtual central reader groups [CRGs] and individual, blinded [‘standard’] readers across the range of UCEIS scores [0–8] represented in the main evaluation set of 40 videos.

The median SD was significantly lower in the blinded, virtual CRG than for ‘standard’ readers [0.54 vs 0.95, respectively; *p* < 0.001]. This was most apparent in videos representing UCEIS scores between 3 and 5, most likely to represent the spectrum mild or moderate endoscopic disease severity [Figure 4], which has substantial implications for clinical trials. The SD was least and also most similar at the extremes of the UCEIS range [≤ 2 and ≥ 6], which implies that the UCEIS has least variance for defining remission or severe endoscopic activity.

### 3.6. Comparison with patient-reported symptoms and established indices

Correlation of UCEIS scores with contemporaneously recorded symptoms and indices ranged from 0.76 for stool frequency [SF, 95% CI = 0.72–0.80] or patient functional assessment [PFA, 95% CI: % 0.71, 0.79], through 0.81 for both stool frequency and rectal bleeding [SF and RB, 95% CI = 0.77, 0.84] to 0.82 for RB [95% CI = 0.78–0.85] and 0.86 for the full MCS [95% CI = 0.83, 0.88 Table 4]. The modified Baron Score consistently correlated less well than the UCEIS with all patient-reported symptoms [Table 4]. It was not possible to compare UCEIS with MCS in this regard, since patient-reported symptoms were derived from data used to calculate the MCS. When endoscopic bleeding was scored 0 on the UCEIS [none], this corresponded to patient-reported RB of 0 or 1 approximately 95% of the time, scored on a 3-point scale [above]. Similarly, when bleeding was scored ≥ 2 on the UCEIS [some free liquid blood to frank bleeding], patients reported RB > 80% of the time.

**Table 4.** Spearman rank correlations of other indices and clinical information with the UCEIS.

Comparator	Correlation [95% CI] with UCEIS	Correlation [95% CI] with Modified Baron Score^a^
Full MCS^+^ [including endoscopy]	0.86 [0.83, 0.88]	N/A^b^
Partial MCS [excluding endoscopy]	0.82 [0.79, 0.85]	0.77 [0.61, 0.88]
Stool frequency subscore	0.76 [0.72, 0.80]	0.73 [0.53, 0.85]
Rectal bleeding subscore	0.82 [0.78, 0.85]	0.74 [0.54, 0.85]
Both stool frequency and rectal bleeding	0.81 [0.77, 0.84]	0.77 [0.58, 0.87]
Patient functional assessment subscore	0.76 [0.71, 0.79]	0.52 [0.24, 0.72]
Modified Baron score^a^	0.78 [0.74, 0.81]	-

Analyses excluded the ‘normal’ and ‘most severe’ videos, as Mayo and PFA data were unavailable, and the common controls videos.

^+^MCS, Mayo Clinic Score; 95% CI, 95% confidence interval.

^a^Modified Baron score as determined by blinded central reader in clinical trials.

^b^N/A, not appropriate since Modified Baron Score from central reader is 1 of the 4 summed quantities in the MCS.

**Figure 5.**
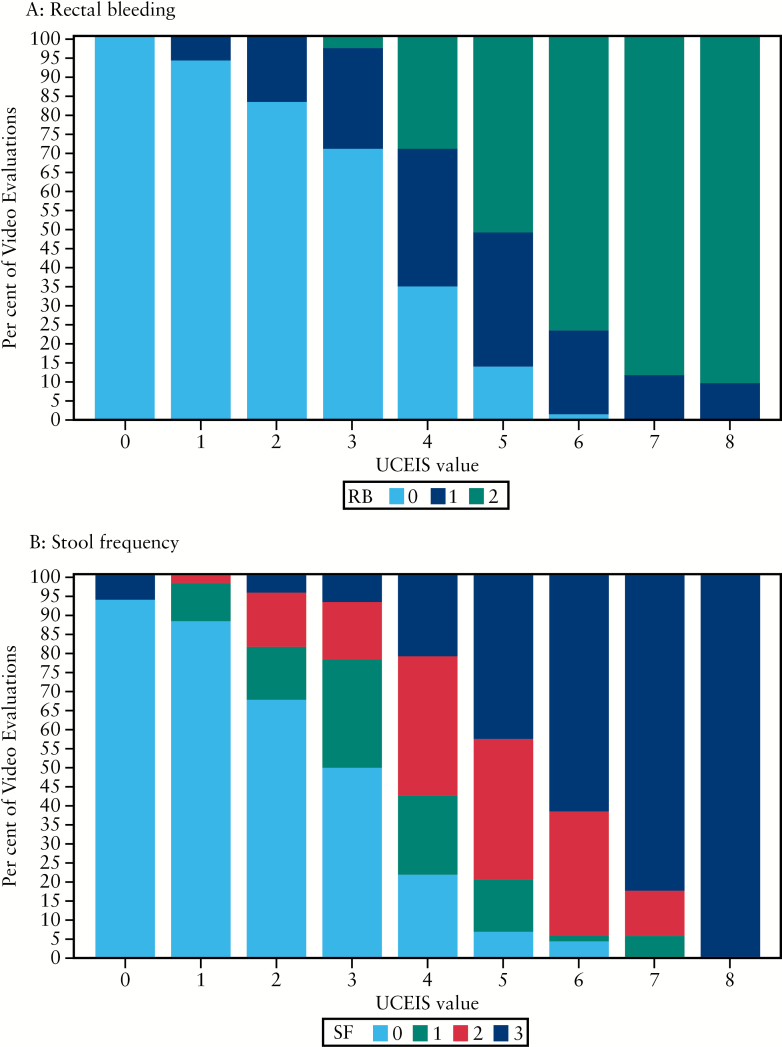
Distribution of rectal bleeding [RB] and stool frequency [SF] items across UCEIS levels. A: When UCEIS ≤ 1, no RB > 95% of the time; when UCEIS ≥ 6, some RB 95% of the time. B: When UCEIS ≤ 1, no increase in SF ~ 90% of the time; when UCEIS ≥ 5, increase in SF 95% of the time.

Scores for RB and SF items showed a clear correlation with endoscopic severity as measured by the UCEIS [Figure 5]. When the UCEIS score was ≥ 5, then RB or an increase in SF was present at least 95% of the time. Such patient-reported symptoms inform clinical thresholds of the UCEIS for evaluating its relationship to outcomes, which are relevant to regulatory assessment.

## 4. Discussion

This study shows that clinical information has minimal impact on endoscopic scoring of disease activity determined by the UCEIS. It also characterises the performance of the UCEIS with regard to patient-reported symptoms and the impact of central reading. In an independent cohort of 40 central readers completing 28 evaluations from a new library of 44 videos, clinical information did not produce a significant change in the UCEIS in any video [0/40]. The UCEIS correlated well with patient-reported symptoms of stool frequency and rectal bleeding [or both], such that when endoscopic bleeding was scored 0 on the UCEIS [none], this corresponded to contemporaneously patient-reported RB of 0 or 1 in 95% of the time. The UCEIS had least variance for defining remission or severe endoscopic activity, but where variance was greatest [mild-moderate activity, UCEIS 3–5], central reading by a group of three readers had most impact. Central reading matters for clinical trial design, whereas the lack of impact of symptoms on endoscopic assessment matters in clinical practice.

The collective performance of the three items accounts for 86% of the variance in the overall assessment of endoscopic severity.^8^ Agreement in scoring of individual items is modest but it is the overall score that matters most, assessed in the most severely affected area at flexible sigmoidoscopy. In this study, the intra- [κ 0.47 to 0.74] and inter-reader [κ 0.40 to 0.50] agreement for the three components of the UCEIS [vascular pattern, bleeding, and erosions and ulcers] was consistent with that reported in phase 3 [intra: κ 0.47 to 0.87; inter: κ 0.48 to 0.54].^8^ Although the impact of training was not quantified in this study, it was considered appropriate as with any descriptive process such as endoscopy reporting. Consistency highlights a strength of the UCEIS in providing a simple, standardised reporting system for the endoscopic appearances of UC and confirms that it is a reliable instrument for assessing endoscopic disease severity.

The influence of clinical information had minimal effect on the variability and reproducibility of UCEIS scoring. No significant differences between readers were demonstrated for any of the items, whether readers were provided with clinical information or not [‘vascular pattern’: κ 0.53 vs 0.50, respectively; ‘bleeding’ κ 0.44 vs 0.40; ‘erosions and ulcers’: 0.47 vs 0.48]. This is internally consistent with the finding that providing unblinded readers with clinical information apparently too severe or too mild for the common control videos did not influence their scoring of the items compared with blinded readers [‘vascular pattern’: 0.66 vs 0.67, respectively; ‘bleeding’ κ 0.56 vs 0.55; ‘erosions and ulcers’: 0.66 vs 0.71]. Although the items of ‘erosions and ulcers’ registered statistical significance [blinded intra-reader κ = 0.47 vs unblinded: κ = 0.74; *p* = 0.047; Table 2], the confidence intervals are wide and consistent with ‘moderate’ to ‘substantial’ agreement whether blinded or unblinded to clinical information when viewing the videos. It will be difficult to improve on an index that [overall] accounts for 86% of variance between readers for the assessment of endoscopic disease activity. A Delphi procedure on the videos with most disagreement might further enhance agreement, but given the range of analyses conducted herein, some of the differences may be chance findings.

The UCEIS correlated well with patient-reported symptoms, including rectal bleeding, stool frequency [or both] and patient functional assessment [rank correlations 0.76 to 0.82]. There were strong correlations between the scores for individual items and overall UCEIS [Figure 5], encouraging application in clinical practice. There was also ‘substantial’ correlation [0.78 to 0.86] with established clinical indices (Mayo Clinic Score [MCS], partial MCS [excluding endoscopy], and modified Baron Score, Table 4). The UCEIS consistently correlated more strongly than the modified Baron score with all patient-reported symptoms. Unfortunately, a comparison between the UCEIS and the MCS for patient-reported symptoms was not possible, since these were derived from data used to calculate the MCS. The impact of patient symptoms on the endoscopic subscore of the MCS is not known. On the other hand, an independent comparative study on responsiveness using clinical trial data suggests that the UCEIS is marginally but consistently more responsive than the MCS.^16^ This may have an advantage in early-phase drug development. It is in this field that a binary endpoint of remission / no remission is inefficient and there is value in assessing relative changes in mean scores.^16,17,18^

This study has shown that variation is both least and most similar for blinded/unblinded groups at the extremes of the UCEIS range [≤ 2 and ≥ 6]. It means that the UCEIS has least variance for defining either remission or severe endoscopic activity. This is an asset when determining responsiveness [the ability to detect change], which was not evaluated in this or previous studies. Relevant to clinical trials, however, is the variance in the mid range [UCEIS 3–5], which was significantly reduced by central reading groups. This was assessed through random groups of three readers, one of whom was ‘experienced’ in central reading, to act as an adjudicator should there be disagreement between ‘standard’ readers, representing normal investigators. Central reading of endoscopy can affect the outcome of clinical trials in mild-moderately active UC. A study of a well-established mesalazine product for mild-moderately active UC did not reach significance compared with placebo [*p* = 0.069].^7^ After blinded central reading of endoscopic videos had excluded 31% of patients for being ineligible for the criterion of a MCS subscore of > 2, the difference between the treatment groups readily achieved significance [29.0% vs 13.8%; *p* = 0.011]. This has implications for a charter on central reading, since the optimal configuration of central reading for scoring is unknown.^9^ Options include an index reader, polling multiple central reader results, adjudicating reads on ‘outliers’, or random selection of videos to be read centrally. The effect of training and revalidation on central readers needs to be determined, since all factors will affect conclusions on drug efficacy and registration.

What still needs to be defined are the levels of the UCEIS for remission, mild, moderate, and severe disease. Taking account of the variance and performance characteristics, the authors speculate that a UCEIS of 0–1 indicates remission and > 6 represents a threshold for severe disease with prognostic implications, although thresholds and their implications need to be defined by prospective study in clinical trials, currently in progress.^17,18^ A score of 0 is likely to become the aspirational goal for both regulatory trials and clinical care;^18^ which should be index independent, since remission is remission. On the other hand, the UCEIS also seems likely to become the favoured instrument for early drug development, when a binary endpoint of remission / no remission is not efficient and there is enormous value in assessing relative changes in mean scores.^16,17,18^ Further limitations of the current study are that the readers in this study may not reflect endoscopists in clinical practice. Readers were selected, trained, and had images available during the rating, so the question of whether clinical information has an impact in real life may be questioned. The answer is training. Although the UCEIS is simple in concept and easy to apply, that 7/47 proposed readers failed to agree with defined interpretations indicates that training is a necessary component of its application, no less for evaluating colitis than for endoscopic polyp detection.

Next steps in the development of the UCEIS include establishing thresholds for remission and severity, and responsiveness to change. It would be valuable to examine how the UCEIS is affected by evaluation of colonic segments at full colonoscopy.^19,20^ On the other hand, caution should be exercised to prevent the UCEIS becoming more complex than necessary. Of greater interest is correlation with histological disease activity or biomarkers, especially for the prognostic value in remission. Central readers can markedly decrease the variability in UCEIS scoring, particularly in the mild-moderate disease spectrum, which is most relevant to clinical trials. The UCEIS is simple to use, derived from the sum of just three items, and accurately accounts for a widest range of disease severity associated with UC, is affected minimally if at all by clinical information, and is ready for practice after appropriate training.

## Conflicts of interest

None declared.

## Author contributions

ST, PK, DS, CB, BY, and WS contributed to the concept and design of the study. DS performed the statistical analysis with further evaluation by JYM and input from DA. PS created the data capture program for video evaluation. PK, BY, and CB coordinated the planning and implementation of the study. ST, MA, JFC, BF, SH, GL, PM, WR, BS, BY, JYM, and WS contributed to the clinical interpretation of the data. All authors contributed to the manuscript.
